# Metabonomics-Based Study of Clinical Urine Samples in Suboptimal Health with Different Syndromes

**DOI:** 10.1155/2013/509134

**Published:** 2013-01-17

**Authors:** Hai-Zhen Cui, Li-Min Wang, Xin Zhao, Yue-Yun Liu, Shao-Xian Wang, Xiao-Hong Li, You-Ming Jiang, Jia-Xu Chen

**Affiliations:** School of Pre-Clinical Medicine, Beijing University of Chinese Medicine, Beijing 100029, China

## Abstract

*Objective*. To explore the urinary biochemistry features of syndromes of traditional Chinese medicine (TCM) such as syndrome of stagnation of liver Qi, spleen deficiency, liver Qi stagnation, and spleen deficiency (LSSDS) in sub-optimal health status (SHS). *Methods*. 12 cases for each syndrome group in SHS were selected, 12 subjects were used as a normal control group, and ^1^H NMR detection was, respectively, carried out, and the data was corrected by the orthogonal signal correction (OSC) and then adopted a partial least squares (PLS) method for discriminate analysis. *Results*. The OSC-PLS (ctr) analysis results of the nuclear overhauser enhancement spectroscopy (NOESY) detection indicated that the syndromes in SHS could be differentiated, and there were significant differences in the levels of metabolites of the urine samples of the four groups; the biomarkers of LSSDS in SHS were found out. The contents of citric acid (2.54 and 2.66), trimethylamineoxide (3.26), and hippuric acid (3.98, 7.54, 7.58, 7.62, 7.66, 7.82, and 7.86) in the urine samples of LSSDS group were lower than that of the normal control group. *Conclusion*. There are differences in the ^1^H-NMR metabolic spectrum of the urine samples of the four groups, and the specific metabolic products of the LSSDS in SHS can be identified from metabonomics analysis.

## 1. Introduction

Since 1984, when the WHO gave the definition to health as paying attention to “the state of complete physical, mental and social well-being” in its “Charter,” the research on the “third state” has been carried out widely at home and abroad. In 1996, scholar Wang from China named this state as “SHS” [1]; thereafter, the study of Suboptimal health domestic started to be involved in many aspects. “Preventive treatment of disease,” namely, the prevention of diseases and maintaining health is the original characteristics and advantages of traditional Chinese medicine. The state of having no “disease” but having the “syndromes” of Suboptimal health has made the current treatment mainly based on the approach of Traditional Chinese Medicine.

It was found by the clinical epidemiological survey that 60%–70% of people in China are in SHS; the incidences of syndrome of stagnation of liver Qi and spleen deficiency among the population of SHS are high and can be up to 17.25 [2], and the incidence of the syndrome in those surveyed on the population of SHS in General Hospitals can be even up to 45.85% [3]. In recent years, metabonomics technology has been widely used in the diagnosis and research of hypertension [4] and other cardiovascular diseases, neurological diseases [5], urinary system diseases [6], diabetes [7], congenital metabolism disorders [8], and cancer [9], and so forth, and it has also been widely used in Chinese herbal compound research [10], safety evaluation of traditional Chinese medicine [11], screening of toxic markers [12], and so on.

Currently combination of syndromes classification and biomedical diagnosis becomes a common model in diagnostics in TCM clinical practice. Clinical treatments of a patient rely on the successful differentiation of a specific TCM syndrome. Our previous studies have shown that [13], through the analysis of the ^1^H NMR spectrum of the plasma of model rats with chronic restraint stress-induced syndrome of stagnation of liver Qi and spleen deficiency, the changes in the spectral peak shape of acetic acid, lactic acid, tyrosine acid, low-density lipoprotein, and the unknown compound at 3.44 ppm were very significant. As a result, the metabonomics method was applied in this study in discussing the syndrome of stagnation of liver Qi and spleen deficiency and assisted in revealing its biological changes. The objective of this study is using metabonomics method to differentiate syndromes with clinical urine samples in Suboptimal health status. 

## 2. Materials and Methods

### 2.1. Source and Grouping of the Subjects

Among the personnel from the Chinese Academy of Sciences who had physical examinations in the Medical Center of Beijing Guanghua Hospital from December 2009 to April 2010, 12 cases of syndrome of stagnation of liver Qi and spleen deficiency, syndrome of stagnation of liver Qi, and syndrome of spleen deficiency in SHS who met the inclusion criteria were, respectively, selected, and 12 cases for the healthy group of normal control were selected. 48 subjects were typical permanent residents in Beijing, and it was shown that the four groups were well balanced on age and sex. Both the research protocol and written informed consent were reviewed and approved by an ethics committee of Beijing University of Chinese Medicine prior to the study initiation. In addition, a TCM syndrome questionnaire for measuring SHS was also used in the study [14], and the SHS was also evaluated as our previously reported [15].

### 2.2. Diagnostic Criteria

#### 2.2.1. Diagnostic Criteria for Suboptimal Health Population

(1) Recurrent physical discomfort and decreased efficiency continued for more than three months due to persistent or excessive fatigue. (2) Have no significant organic diseases or psychological disorders.

Only those who meet both the above two criteria could be diagnosed as Suboptimal health population.

#### 2.2.2. Inclusion Criteria

(1) Meet the diagnostic criteria. (2) Age should be between 18 and 49 years old. (3) Informed consent: all subjects should have a consent signed that demonstrates an understanding of this study.

Only those who meet all the above three criteria could be included.

#### 2.2.3. Exclusion Criteria

(1) Subjects who do not meet the inclusion criteria. (2) Women who are pregnant, in lactation period or are attempting to get pregnant. (3) Subjects who do not sign the informed consent form. (4) Subjects diagnosed with metabolic syndrome or who have received any treatment.

#### 2.2.4. Diagnostic Criteria for the Syndromes

For the diagnosis of syndrome of stagnation of liver Qi, and spleen deficiency, syndrome of stagnation of liver Qi and syndrome of spleen deficiency, please refer to “GB/T16751.2-1997, Clinic terminology of traditional Chinese medical diagnosis and treatment-Syndromes” of the People's Republic of China (Bureau of Technical Supervision, implemented on October 1, 1997).

## 3. Methods

### 3.1. Instruments and Reagents

*Instruments* are INOVA 600 MHz superconducting NMR spectrometer (Varian Inc., USA), equipped with pulsed field gradient and a three-resonance gradient probe; Centrifuger: produced by Eppendorf MiniSpin Plus, Germany.

*Reagents* are deutoxide (D2O), produced by Cambridge Isotope Laboratories Inc., USA, 99.9%; 2,2,3,3,-d(4)-3-Trimethylsilyl) propionic acid sodium (TSP), purchased from Merck, Germany.

### 3.2. Sample Preparation before NMR Experiment [16, 17]

About 5 mL of middle-segment fasting morning urine sample of the subjects were collected. Then the samples were centrifuged at 3500 rpm for 10 min after collection. Then 1 mL of the supernatant was taken and placed in the refrigerator with temperature at −20°C for cryopreservation. 30 *μ*L of TSP/D_2_O (1 mg/mL) was added into the 5 mm NMR tube. After the urine samples were thawed at room temperature, 350 *μ*L of phosphate buffer solution and 350 *μ*L of the urine were added into the EP tube, after being well mixed by shaking, centrifuged at 13,000 rpm for 10 min, and 600 *μ*L of the supernatant was added into the NMR tube mentioned above and mixed and was ready for use.

### 3.3. NMR Data Collection

Detection was carried out in the Magnetic Resonance Laboratory of the Medical Center of Biomedical Analysis of National Academy of Military. Under the conditions of 27°C, Carr-Purcell-Meiboom-Gill (CPMG) and longitudinal eddy delay (LED) were, respectively, adopted for the experiment on the VARIAN UNITYINOVA 600 MHz Superconducting Fourier Transform NMR spectrometer, the presaturated method was used to inhibit the water peak, the saturation time was 2 s, spectral width was 8000 Hz, the sampling point was 32 k, and it accumulated 64 times. The presaturation frequency and central frequency were both at the position of water peak. The free induction decay (FID) signal was converted to NMR spectrum through the Fourier transform at the 128 k point. The peak of the chemical shift of TSP was used as a reference position and set to 0 ppm.

The obtained NMR data was converted to a spectrum by the Fourier transform, after phase and baseline correction, the spectrum within a certain range was piece-wisely integrated at a width of 0.04 ppm for each segment, and the segments with solvent peaks and urea peaks (4.6 to 6.2) were excluded at the same time. The integral range of the NOESY data was 0.4 ppm ~ 9.4 ppm. The integral was normalized by the total integral intensity of each spectrum. The obtained data was output and converted to an Excel file and saved.

### 3.4. NMR Data Analysis

All the integral data was output to Excel files and saved and then entered into the SIMCA-P + software (V10.04, Umetrics, Umeå, Sweden) for analysis. Orthogonal signal corrections (OSC) were performed first, and PLS analysis was then conducted. The analysis results were expressed as scores plot and loadings plot. The degree of separation for each group of data was determined from the scores plot, and the metabolites that contributed to the composition were obtained from the factor loadings plot.

## 4. Results

### 4.1. Typical ^1^H-NMR Spectra of the Samples of Four Groups (See Figure 1)

### 4.2. Results of the Overall Comparative Analysis of the Four Groups

**Figure 2:**
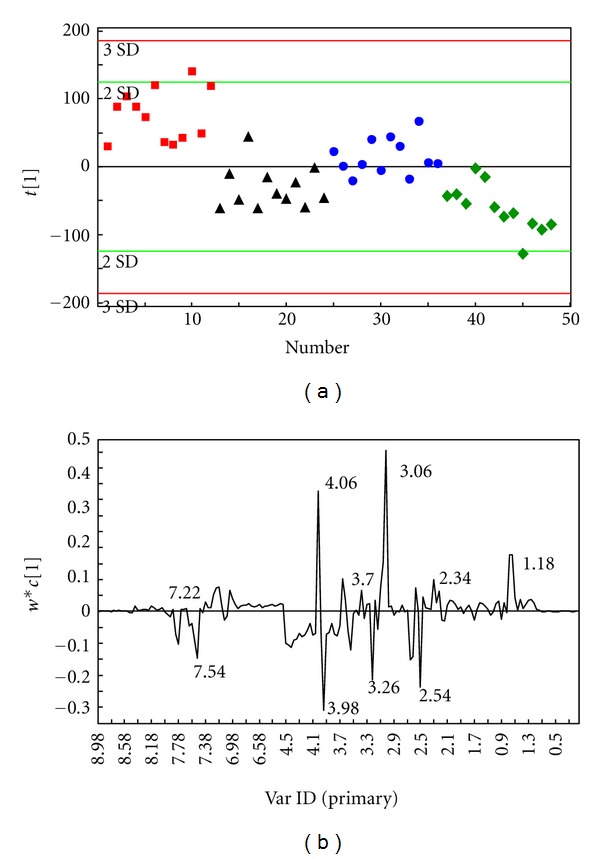
(a) The scores plot of the OSC-PLS (ctr) analysis of the NMR (NOESY) of the urine samples of the four groups: A (■), B (●), C (◆), and D (▲). (b) The loadings plot of the OSC-PLS (ctr) analysis of the NMR (NOESY) of the urine samples of the four groups.

The OSC-PLS analysis results of ^1^H-NMR spectrums of the four groups—syndrome of stagnation of liver Qi and spleen deficiency (group A), syndrome of stagnation of liver Qi (group B), syndrome of spleen deficiency (group C), and the normal control (group D)—are shown in Figure 2.

Figure 2(a) is a one-dimensional scores plot obtained by PLS analysis after OSC denoising processing. We can see that the four groups of samples are distinguishable from each other, and there are no overlap and duplication among the four groups. The results indicate that there are differences in the metabolic phenotypes of the four groups, and the intergroup differences are significant. The group of syndrome of stagnation of liver Qi and spleen deficiency (Group A) shows obvious separation trends from the group of normal control (group D) and group of syndrome of spleen deficiency (Group C) along the *t*_1_ axis; it can be said that the group of syndrome of stagnation of liver Qi (Group B) and the group of syndrome of spleen deficiency (Group C) show rough separation trends along the *t*_1_ axis.

The abscissa of Figure 2(b) corresponds to the chemical shift, wherein the positive values on the vertical axis represent that, within the chemical shift range, the corresponding metabolite concentrations in most samples of the syndrome of stagnation of liver Qi and spleen deficiency are larger; the negative values represent that, within the chemical shift interval, as compared with the group of normal control and the group of the syndrome of spleen deficiency, the metabolite concentrations in the group of syndrome of stagnation of liver Qi and spleen deficiency are lower. The specific metabolites should be analyzed in combination with the intergroup pairwise comparison results.

As the diet and lifestyle of the subjects in this study were not strictly restricted, in order to improve the classification results, intergroup pairwise comparisons were using the PLS-DA analysis which was carried out after OSC denoising processing.

### 4.3. The Analysis Results of the Comparison between the Group of Syndrome of Stagnation of Liver Qi and Spleen Deficiency and the Group of Normal Control

**Figure 3:**
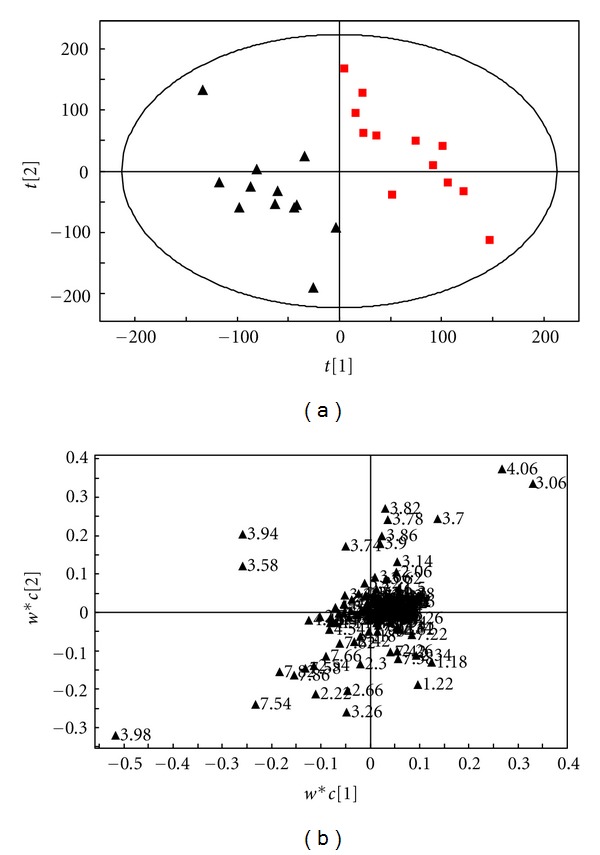
(a) The scores plot of the OSC-PLS (ctr) analysis of the ^1^H-NMR (NOESY) spectrum of the urine samples from the group of syndrome of stagnation of liver Qi and spleen deficiency (■) and the group of normal control (▲). (b) The loadings plot of the OSC-PLS (ctr) analysis of the ^1^H-NMR (NOESY) spectrum of the urine samples from the group A and group D.

The OSC-PLS analysis results of the ^1^H-NMR spectrum of the urine samples of the group of syndrome of stagnation of liver Qi and spleen deficiency (Group A) and the group of normal control (group D) are shown in Figure 3. It can be seen from the scores plot (Figure 3(a)) that these two groups of samples can be well separated along the *t*_1_ axis, with no overlap and duplication. This indicates that the two groups have differences in the metabolic phenotypes, and the intergroup difference is significant. We can see from the loadings plot (Figure 3(b)) that most points are concentrated near the origin, and only a few points are away from the origin, which represent the compounds that resulted in the differences between the two groups. We can find out the chemical shifts of the metabolites NMR peak values with their differences by combining the loadings plot with the primary spectrum, and the metabolites with differences of these two groups of urine samples are shown in Table 1.

### 4.4. The Analysis Results of the Comparison between the Group of Syndrome of Stagnation of Liver Qi and Spleen Deficiency and the Group of Syndrome of Stagnation of Liver Qi

**Figure 4:**
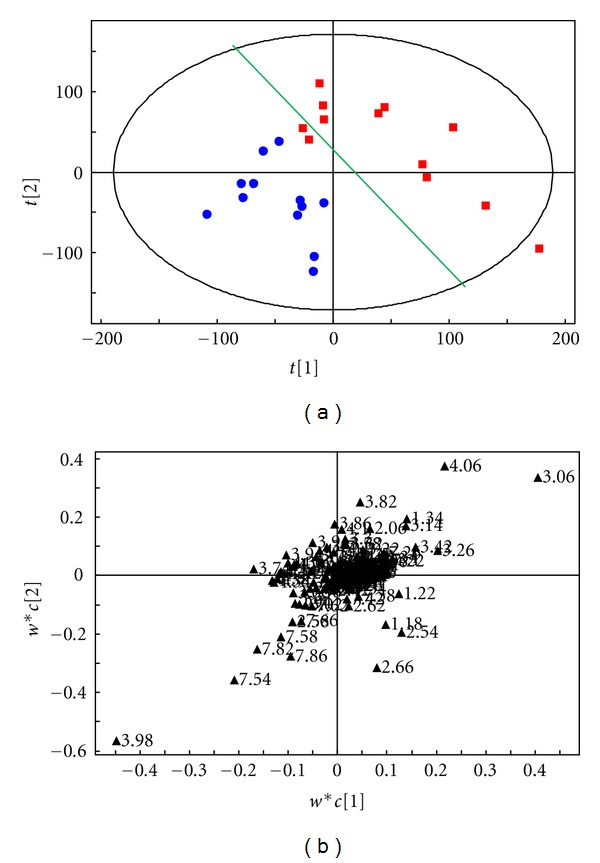
(a) The scores plot of the OSC-PLS (ctr) analysis of the ^1^H-NMR (NOESY) spectrum of the urine samples of the group of syndrome of stagnation of liver Qi and spleen deficiency (■) and the group of syndrome of stagnation of liver Qi (●). (b) The loadings plot of the OSC-PLS (ctr) analysis of the ^1^H-NMR (NOESY) spectrum of the urine samples from group A and group B.

The OSC-PLS analysis results of the ^1^H-NMR spectrum of urine samples of the group of syndrome of stagnation of liver Qi and spleen deficiency (Group A) and the group of syndrome of stagnation of liver Qi (Group B) are shown in Figure 4. It can be seen from the scores plot (Figure 4(a)) that these two groups can be roughly separated by the green line as indicated, the vertical to the green line representing the intergroup differences, and the parallel to the green line representing the intra-group differences. We can see from the loadings plot (Figure 4(b)) that most points are concentrated near the origin, and only a few points are away from the origin, which represent the compounds that resulted in the differences between the two groups. We can find out the chemical shifts of the metabolites NMR peak values with their differences by combining the loadings plot with the primary spectrum, and the metabolites with differences of these two groups of urine samples are shown in Table 1.

### 4.5. The Analysis Results of the Comparison between the Group of Syndrome of Stagnation of Liver Qi and Spleen Deficiency and the Group of Syndrome of Spleen Deficiency

**Figure 5:**
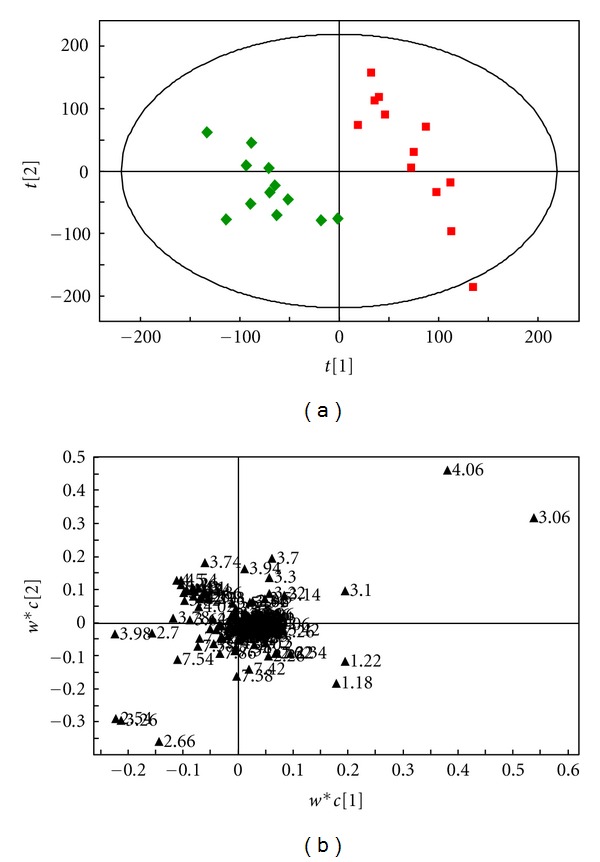
(a) The scores plot of the OSC-PLS (ctr) analysis of the ^1^H-NMR (NOESY) spectrum from group A (■) and group C (◆). (b) The loadings plot of the OSC-PLS (ctr) analysis of the ^1^H-NMR (NOESY) spectrum of urine samples from group A and group C.

The OSC-PLS analysis results of the ^1^H-NMR spectrum of the urine samples of the group of syndrome of stagnation of liver Qi and spleen deficiency (Group A) and the group of syndrome of spleen deficiency (Group C) are shown in Figure 5. It can be seen from the scores plot (Figure 5(a)) that these two groups of samples can be well separated along the *t*_1_ axis, with no overlap and duplication, indicating that the two groups have differences in the metabolic phenotypes, and the intergroup difference is significant. We can see from the loadings plot (Figure 5(b)) that most points are concentrated near the origin, and only a few points are located away from the origin, which represent the compounds that resulted in the differences between the two groups. We can find out the chemical shifts of the NMR peak values of the metabolites with its differences by combining the loadings plot with the primary spectrum, and the metabolites with its differences of these two groups of urine samples are shown in Table 1.

### 4.6. Summary of the Analysis Results of the Urine Samples from the Four Groups

Based on the results above, there are certain differences among the samples from the four groups. Further analysis of the loadings plots of the principal component by intergroup comparison of the groups showed that the following substances (Table 1) contributed greatly to the classification, namely, the potential markers.

## 5. Discussion

In this study, the PCA analysis and the PLS analysis which were carried out after OSC denoising processing were comprehensively used, and the results were expressed by scores plots, and loadings plots. Wherein, the scores plots were plotted using the principal component as the axis, reflecting the differences between the classes, to access classification information of the samples. The loadings plots reflected the variables and their contributions to classification. By analyzing the scores plots, loadings plots and the primary spectrums of the intergroup pairwise comparison, some biomarkers were obtained. The potential biomarkers of syndrome of stagnation of liver Qi and spleen deficiency should be the metabolites that showed changes in content as compared with the group of normal control. Furthermore, there also should be changes shown in the content compared with the syndrome of stagnation of liver Qi and the syndrome of spleen deficiency, and the metabolites meeting this criterion are as follows: creatinine (3.06 and 4.06), trimethylamine oxide (3.26), and hippuric acid (3.98, 7.54, 7.58, 7.62, 7.66, 7.82, and 7.86).

Syndrome of stagnation of liver Qi and spleen deficiency is one of the common syndromes of SHS and mental disorders in TCM clinic [18], our recently studies shown: the Xiaoyaosan (XYS) decoction reversed chronic stress induced decreases in brain-derived neurotrophic factor (BDNF) and increases in tyroxine hydroxylase (TrkB), and neurotrophin 3 (NT-3) in the frontal cortex and the hippocampal CA1 subregion [19]. The xiaoyaosan decoction can significantly downregulate the contents of leptin and leptin receptor (*ob-R*) at the arcuate nucleus (ARC) in the basal of hypothalamus in the hypothalamus of chronic stressed rats [20]. Meanwhile, the XYS-decoction-containing serum is significant in improving mitochondrial membrane potential and apoptotic rate of hippocampus neuron induced by oxidative-stress [21]. Excessive pressure from work or a poor emotion state will lead to the dysfunction of “liver controlling conveyance and dispersion,” and the stagnation of liver Qi will result in spleen and stomach disorders. As a result, fatigue and other symptoms will occur.

Creatinine is a metabolite produced during metabolism of muscle tissue and is also a compound related to the renal function. Therefore, the relative increase in creatinine levels suggests the decrease in the metabolic function of syndrome of stagnation of liver Qi and spleen deficiency in SHS. Trimethylamine oxide (TMAO) has a role in promoting the growth of muscle tissue, and the decrease in the content of trimethylamine oxide suggests the decline of the “spleen governing muscles” function of the syndrome of stagnation of liver Qi and spleen deficiency in SHS. Hippuric acid is a small molecule urine toxin, and there is a study indicating that hippuric acid can accurately reflect the energy metabolism of the liver and any reason affecting the oxidative and phosphorylation ability of mitochondria of liver cells; thus, resulting in hepatic energy metabolism disorders can affect the synthesis ability of hippuric acid [22]. Therefore, the changes in hippuric acid also demonstrate that the energy metabolism in the liver of syndrome of stagnation of liver Qi and spleen deficiency in SHS has been damaged to a certain extent.

In this study, we analyzed the metabolic phenotype spectrums of the urine samples of the syndrome of stagnation of liver Qi and spleen deficiency, syndrome of stagnation of liver Qi, syndrome of spleen deficiency in SHS, and the group of normal control from the perspective of metabonomics, and there were significant differences in the metabolites for the syndromes in SHS. These differences are based on the changes in the metabolism of different substances or the metabolic network. The metabonomics technology may be used to identify the specific metabolites for the biological basis of the syndromes of TCM, and the methods of bioinformatics can also be used to analyze the functions of the biomarkers, and to determine the “Metabolic spectrum related to syndrome of TCM” [13].

We also describe in detail the metabolic consequences of syndromes in SHS. In particular, we have shown that the specific metabolic products of the syndrome of stagnation of liver Qi and spleen deficiency in SHS can be identified from metabonomics analysis: creatinine (3.06 and 4.06), trimethylamine oxide (3.26), and hippuric acid (3.98, 7.54, 7.58, 7.62, 7.66, 7.82, and 7.86). We consider the results of this study to add considerably to our understanding of the biochemistry of TCM syndromes.

## Figures and Tables

**Figure 1:**
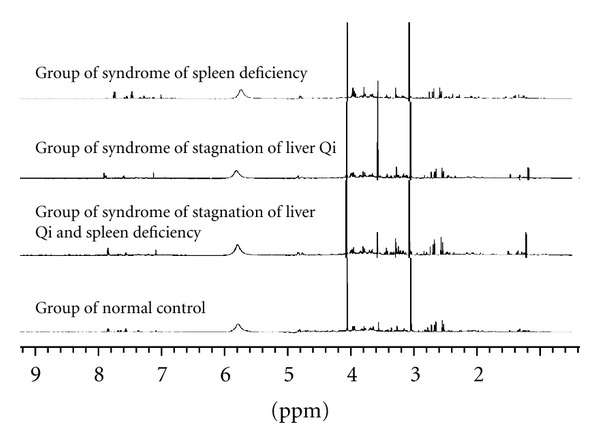
Typical ^1^H-NMR spectra of the samples of four groups.

**Table 1:** The lookup table of concentration changes of the relevant metabolites.

Chemical shifts (ppm)	Compound name	Group A versus group D	Group A versus group B	Group A versus group C
1.34, 4.14	Lactic acid	—	↑	—
2.54, 2.66	Citric acid	—	—	↓
3.06, 4.06	Creatinine	↑	↑	↑
3.26	Trimethylamine oxide	↓	↑	↓
3.26, 3.42	Taurine	—	↑	—
3.98, 7.54, 7.58, 7.62, 7.66, 7.82, 7.86	Hippuric acid	↓	↓	↓

↑ indicates relatively high levels of contents; ↓ indicates the relatively low levels of contents; — indicates that contents are similar to each other. TMAO: trimethylamine oxide.
